# Effectiveness of Pharmaceutical Counseling in Sore Throat Management According to Patients and Pharmacists

**DOI:** 10.3390/healthcare13212708

**Published:** 2025-10-27

**Authors:** Piotr Merks, Sebastian Sikorski, Urszula Religioni, Dariusz Świetlik, Katarzyna Plagens-Rotman, Ewelina Drelich, Justyna Kaźmierczak, Aneta Krolak-Ulińska, Radosław Sierpiński, Zbigniew Doniec

**Affiliations:** 1 Department of Pharmacology and Clinical Pharmacology, Faculty of Medicine, Collegium Medicum, Cardinal Stefan Wyszyński University in Warsaw, 01-938 Warsaw, Poland; piotrmerks@googlemail.com; 2 Department of Administrative Law and Local Government, Cardinal Stefan Wyszyński University in Warsaw, 01-938 Warsaw, Poland; 3 School of Public Health, Centre of Postgraduate Medical Education, 01-826 Warsaw, Poland; 4 Division of Biostatistics and Neural Networks, Medical University of Gdańsk, 80-210 Gdańsk, Poland; 5 Center for Pediatric, Adolescent Gynecology and Sexology, Division of Gynecology, Department of Gynecology, Poznan University of Medical Sciences, 61-701 Poznan, Poland; 6 The Polish Pharmacy Practice Research Network (PPPRN), 00-238 Warsaw, Poland; 7 Anesthesiology and Intensive Care Unit, Węgrów Regional Hospital, 07-100 Węgrów, Poland; 8 Faculty of Medicine, Collegium Medicum, Cardinal Stefan Wyszynski University, 01-938 Warsaw, Poland; 9 Medical Institute, Academy of Applied Sciences, 33-300 Nowy Targ, Poland

**Keywords:** pharmaceutical counseling, community pharmacy, pharmaceutical care, effectiveness evaluation, antibiotic therapy

## Abstract

**Background:** Primary care overload, limited access to physicians, and rising antimicrobial resistance highlight the role of pharmacists in managing minor ailments such as sore throats. We evaluated pharmacy-based counseling in Poland supported by point-of-care testing and symptomatic therapy. **Methods:** Multicenter, prospective observational study across 23 community pharmacies. Adults (≥18 years) with sore throat underwent group A streptococcus (GAS) rapid antigen testing. Patients with a positive test result were referred to physicians, while others received pharmacist counseling and ketoprofen throat spray. Standardized questionnaires captured symptom severity, perceived effectiveness, onset/duration, convenience, adherence, and patient-reported outcomes. **Results:** 142 patients were included. Among ketoprofen users, 98.4% reported improvement, and 75% rated relief ≥8/10. Compared with prior remedies, 88.3% judged ketoprofen more effective, and 86.7% reported faster onset. The spray was convenient for 91.4% of participants; no overdosing occurred. Qualitative feedback emphasized rapid relief, easier swallowing/speaking, and return to daily activities without physician consultation. **Conclusions:** Polish community pharmacy practice, an integrated sore throat pathway combining point-of-care RADT with structured pharmacist counseling and symptomatic treatment, was feasible, acceptable, and without notable safety concerns. As a pilot, these practice-based findings warrant larger comparative and economic studies to confirm clinical effects and assess potential impact on antibiotic use.

## 1. Introduction

Sore throat is among the most frequent reasons for patients to seek medical or pharmaceutical assistance [1]. It may result from a variety of causes, including viral or bacterial infections, allergic reactions, or mechanical and chemical irritation. Although the majority of cases do not require antibiotic therapy, inappropriate and frequent use of antibiotics remains common in clinical practice [2], thereby contributing to the global challenge of antimicrobial resistance. This situation underscores the need for rational approaches that combine accurate diagnostics with safe and effective symptomatic management.

In this context, the role of pharmacists as first-line healthcare professionals has been steadily expanding. Their responsibilities extend beyond the traditional dispensing of medications to encompass educational, preventive, and diagnostic activities. One of the tools that supports this broader scope of practice is the rapid group A streptococcus (GAS) test (commonly referred to as the streptotest), which enables differentiation between viral and bacterial infections [3,4,5]. Based on test outcomes, pharmacists are able to advise patients on appropriate next steps, including referral to a physician when antibiotic therapy is indicated or recommendation of symptomatic treatment in cases of viral etiology.

International experience demonstrates that pharmacy-led interventions in the management of minor ailments can generate substantial benefits for healthcare systems. Services such as sore throat test-and-treat programs, implemented, for example, in the United Kingdom, Wales, and Canada, have been shown to reduce unnecessary antibiotic prescribing, improve patient access to timely care, and increase patient satisfaction [4,5]. In many developed countries, community pharmacies already play a significant role in relieving the burden on primary care by facilitating earlier intervention, decreasing the need for unnecessary general practitioner consultations, and supporting antimicrobial stewardship. This system not only enhances patient comfort but also contributes to a more efficient allocation of healthcare resources.

In Poland, the role of the pharmacist in this area is still emerging. Experiences associated with the implementation of new pharmacy services are particularly valuable for informing systemic changes, including the development of standardized procedures, educational strategies, and regulatory frameworks. Studies assessing both clinical outcomes and patient experiences are therefore essential to guide future expansion of pharmaceutical care.

One example of symptomatic management is the use of ketoprofen in spray formulation, a non-steroidal anti-inflammatory drug with local action. This preparation provides rapid analgesic effects and represents an alternative to systemic medications, thereby offering additional value in viral sore throat cases where antibiotics are not required. The spray form also improves ease of administration and enables targeted delivery to the site of inflammation, enhancing patient acceptance and adherence. Importantly, while ketoprofen is used in this study as a model intervention, the underlying approach, pharmaceutical counseling supported by point-of-care diagnostic testing and evidence-based symptomatic treatment, may be generalized to other non-antibiotic therapies available in community pharmacies.

Against this background, our contribution focuses on the implementation perspective for Poland. To our knowledge, this is the first multicenter, prospective real-world study in the country that combines RADT (the rapid strep test, streptotest) with structured pharmacist counseling and parallel assessment of patient- and pharmacist-reported outcomes. The study provides real-world evidence on feasibility, safety, dosing adherence, and acceptability of the aerosol formulation, evidence that is crucial for implementing pharmacy services and for regulatory and payment decisions in health-care systems organized similarly to Poland’s. Accordingly, the present study aimed to evaluate the effectiveness of pharmacist-led sore throat management in community pharmacies in Poland, with particular emphasis on integrating streptotest into a structured counseling pathway and on documenting patient and pharmacist-reported outcomes.

## 2. Materials and Methods

The study was conducted between January and March 2025 in 23 community pharmacies across Poland and employed a prospective, observational design. Its primary objective was to evaluate the effectiveness of pharmaceutical counseling provided to patients presenting with sore throat, with particular focus on the application of a rapid diagnostic test for group A streptococcus (streptotest) and the subsequent recommendation of symptomatic treatment. In addition, the study assessed both patient and pharmacist perspectives regarding the quality, relevance, and perceived effectiveness of the counseling in order to gain deeper insights into the impact of pharmaceutical care on symptom management and patient satisfaction.

Eligible participants were adults (≥18 years) whose primary complaint was sore throat. Further inclusion criteria comprised the ability to attend two visits at the same pharmacy and provision of written informed consent. All data were collected anonymously, in accordance with ethical standards and current data protection regulations.

The study procedure consisted of two stages. During the first visit, the pharmacist conducted a structured interview, assessed symptoms, and performed the streptotest to differentiate between bacterial and viral infections. Patients with a positive test result (14) were referred to a physician, whereas those with negative results received symptomatic treatment recommendations involving over-the-counter products. In cases where ketoprofen was selected, patients were invited to continue participation in the survey. At the second visit, participants returned a completed questionnaire assessing the perceived effectiveness of treatment as well as the quality of the pharmaceutical counseling received. These data formed the basis for the evaluation of the effectiveness and acceptability of pharmaceutical interventions in the management of sore throat.

Primary outcomes (in the RADT-negative cohort) included: (1) patient-reported improvement following counseling and symptomatic treatment (yes/no) and (2) the amount of symptom relief at follow-up, rated on a 0–10 scale (0 = no relief, 10 = complete relief); baseline pain intensity (0–10) was recorded at the first visit.

Secondary outcomes included: perceived effectiveness compared to previously used agents (0–10; higher = more effective), rapid onset of action compared to previous formulations (0–10; higher = faster), convenience/acceptability of the formulation (0–10), adherence to recommendations (including information on possible overdosing: yes/no), and ability to return to daily activities (yes/no, with an open-ended comment).

Safety included monitoring for adverse events associated with the recommended therapy and dosing errors/overuse (yes/no).

### 2.1. Research Tool

The survey questionnaire used in the study was developed specifically for the project. It consisted of two parts:

- Part 1 (completed by the pharmacist during Visit 1): Included questions about the patient’s age and gender, symptom description (e.g., difficulty swallowing or speaking), streptotest result, and therapeutic decision.
- Part 2 (completed by the patient after using the medication): Included questions on perceived improvement, level of pain reduction (on a 0–10 scale), onset of therapeutic effect, comparison with previously used products, convenience of the spray form, and adherence to dosing recommendations.

The questionnaire was standardized and included closed-ended questions (e.g., rating scales) as well as a few semi-open questions allowing for additional comments.

### 2.2. Patient Flow

A total of 142 patients presenting to community pharmacies with sore throat symptoms were enrolled in the study. All participants underwent a streptotest and received an initial pharmaceutical consultation. In the majority of cases, the test result was negative (128), indicating no need for antibiotic therapy and eligibility for symptomatic management with over-the-counter medication. In this study, ketoprofen in spray form was recommended as the treatment option, and pharmacists provided patients with detailed guidance on its use as well as instructions for further self-management. After several days, participants returned completed questionnaires, which were subsequently used to assess treatment effectiveness and to evaluate the quality of the pharmaceutical counseling provided. Patients with positive streptotest (14) results were excluded from further analysis and referred to a physician for appropriate medical care.

### 2.3. Ethical Aspects

The study was approved by the Bioethics Committee of the Poznan University of Medical Sciences (Resolution No. 666/2024 of 9 October 2024).

### 2.4. Data Analysis

All statistical calculations were performed using StatSoft, Inc., Tusla, OK, USA, STATISTICA (data analysis software system), version 12.0, www.statsoft.com and Microsoft Excel. Quantitative variables were described using the arithmetic mean, standard deviation, median, minimum and maximum values (range), and 95% confidence intervals (95% CI). Qualitative variables were presented as counts and percentages (%).

## 3. Results

The relevant part of the study included 128 adult patients (aged ≥18 years) who presented to one of 23 community pharmacies in Poland with complaints of sore throat. All participants were informed about the purpose and nature of the study, gave written informed consent, and met the inclusion criteria. Pharmacists performed a group A β-hemolytic streptococcus (Streptococcus pyogenes) rapid antigen test, and in the case of a negative result, provided pharmaceutical counseling and recommended symptomatic treatment with ketoprofen in spray form.

Characteristic of the study group is presented in Table 1.

### 3.1. Severity of Sore Throat and Need for Intervention

Prior to treatment, respondents rated the severity of their sore throat at an average of 6.6 points on a 10-point scale (0—no pain, 10—maximum pain). The largest group of patients (60.9%) reported severe pain (8 points or more). Moderate pain (5–7 points) was reported by 35.9% of respondents, while only 3.2% were classified as experiencing mild symptoms (0–4 points). This distribution indicates that most patients seeking pharmaceutical advice were experiencing significant discomfort, justifying the need for effective and rapid therapeutic intervention.

Regarding the pharmacists’ assessment, throat swelling was most often classified as moderate in 65 cases (50.8%), and severe in 31 cases (24.2%). Redness, also, was most often classified as moderate in 69 cases (53.9%), and severe in 35 cases (27.3%) (Table 2).

### 3.2. Effectiveness of Symptomatic Treatment with Ketoprofen

During treatment, patients were asked to evaluate the effectiveness of the product in relieving symptoms. Analysis showed that 98.4% of patients reported improvement, with 50% indicating significant symptom relief (ratings of 9–10), and another part reporting moderate improvement (ratings of 6–8). Only 2 individuals did not notice clear improvement after using the product.

Regarding the degree of sore throat relief, 75% of patients (96 individuals) gave ratings of 8–10 points, indicating significant or complete symptom resolution. The most frequently chosen rating was “10”—complete relief—reported by 31 patients (24.2%).

### 3.3. Comparison with Previously Used Products

Participants were also asked to compare the effectiveness of ketoprofen with other products they had previously used to treat sore throats. The vast majority (88%) considered ketoprofen to be more effective than previously used remedies. Notably, 52 patients gave the highest scores (9–10), describing it as “much better” than other treatments. Only one person rated the product as less effective than alternatives (Table 3).

### 3.4. Speed and Duration of Action

Another aspect assessed was the speed of onset of therapeutic effect. Almost 87% of patients reported that relief occurred faster compared to other medications they had used. Fifty individuals gave scores of 8–10, indicating maximum perceived speed of action (Table 4).

Regarding the degree of pain relief provided by ketoprofen, 75% of patients rated the effect as significant. Another 25% reported moderate pain relief, and no respondents indicated a lack of improvement (Table 5).

All participants adhered to the recommended dosing regimen (Table 6).

### 3.5. Assessment of Administration Form and Ease of Use

Ketoprofen in spray form was rated as highly convenient to use: 91.4% of patients (117 individuals) assigned a score between 6 and 10, with 41 patients (32.0%) giving the maximum score of “10.” Only 2 patients considered the spray inconvenient (ratings 0–3). The average convenience score was 8.3, reflecting a generally positive reception of this form of treatment.

### 3.6. Adherence to Dosing and Safety of Use

All patients followed the dosing instructions provided. No cases of exceeding the maximum daily dose were recorded. This result may be interpreted as evidence of effective pharmaceutical communication and the clarity and comprehensibility of the pharmacist’s instructions during the consultation.

### 3.7. Patient Comments on Product Effectiveness

In the open-ended comments, patients expressed positive opinions about the speed of action and ease of use of the product, particularly the absence of a need to swallow a tablet and the neutral taste. Many reported feeling better after the first use, and stated that the treatment allowed them to return more quickly to work or daily responsibilities without the need for a doctor’s consultation. For some, the use of the product also improved comfort when speaking and swallowing, which had previously been impaired.

### 3.8. Patient Satisfaction with the Service

Above 98% of patients reported improvement and satisfaction with the service, with 53.6% reporting “significant” satisfaction. The preferred payer is the public sector—primarily the public payer (96.9%), followed by the Ministry of Health (50.8%) and the Social Insurance Institution (36.7%); patients themselves (7%) or supplementary insurance (4.7%) were less frequently indicated. Most accept fees > PLN 100 (61.3%), with significantly lower rates for lower price ranges (Table 7).

## 4. Discussion

The results of this study confirm the practice-based effectiveness of pharmaceutical counseling provided to patients presenting to community pharmacies with sore throat complaints. The implementation of a structured diagnostic and therapeutic protocol— including a group A streptococcus rapid antigen test, evaluation of the need for antibiotic therapy, and recommendation of symptomatic treatment—proved to be safe, well accepted, and associated with a high rate of patient-reported improvement. As many as 98% of patients reported relief, and more than 75% described significant symptom reduction. These findings highlight not only the potential benefits observed in this project, but—more importantly—the clinical appropriateness of pharmacist-led assessment in minor ailment management [3]. Importantly, no adverse events [6] or cases of inappropriate dosing were observed, suggesting both a high level of safety and effective patient–pharmacist communication.

To address coherence explicitly: while ketoprofen in spray form was used here as an illustrative symptomatic option, our primary object of evaluation was the integrated pharmacy pathway. The ketoprofen results should therefore be read as patient-reported outcomes embedded in that pathway, not as a stand-alone product efficacy trial. In this broader context, similar benefits may be expected with other evidence-based over-the-counter options for sore throat management when supported by appropriate pharmaceutical counseling. Thus, the novelty of this work lies in demonstrating the feasibility and acceptability of a scalable pharmacy-based model (rapid diagnostic testing combined with structured counseling and non-antibiotic symptomatic therapy) rather than in establishing superiority of a single product. This approach represents a reproducible framework for community pharmacies, with potential applications beyond sore throat management.

The comparative evaluation against previously used remedies revealed a clear advantage of the pharmacy-based intervention. Nearly 90% of patients found the recommended treatment more effective, and over 86.7% reported faster onset of action. Although these perceptions relate to ketoprofen, they indirectly support the added value of pharmacist-guided therapy selection within a structured consultation. The findings underline the importance of both pharmacological properties (e.g., analgesic and anti-inflammatory activity of a non-steroidal anti-inflammatory drug) and pharmaceutical formulation (spray application), which influence acceptability and adherence. In our setting, these effects occurred alongside standardized counseling, indicating that the observed benefits reflect the integrated pathway rather than a product-only effect.

These observations support the growing role of pharmacists as first-contact healthcare providers in the management of minor ailments [7]. In the context of primary care overload and shortages of physicians, pharmacists can effectively assume responsibility for triaging and managing conditions such as sore throat, which in most cases are viral and do not require antibiotic therapy [4,8]. In addition to improving patient outcomes, this model contributes to reducing unnecessary medical consultations and supporting antimicrobial stewardship, thereby enhancing efficiency in the healthcare system.

The inappropriate use of antibiotics remains a critical global challenge, fueling the rise in antimicrobial resistance [9,10]. Practices such as prescribing antibiotics “just in case” or under patient pressure accelerate the emergence of resistant strains and reduce therapeutic options for more severe infections [11,12]. In this regard, streptotests performed in pharmacies may represent a valuable public health intervention. As shown in this study, the test is feasible for pharmacists to administer, acceptable to patients, and effective in guiding decision-making [13,14,15]. Importantly, previous evidence suggests that patients are more likely to accept non-antibiotic recommendations when a diagnostic test confirms the absence of bacterial infection.

Our results are consistent with the international literature, which demonstrates the effectiveness of pharmacy-led services in the management of minor ailments. In countries such as the United Kingdom, Canada, Germany, and the Netherlands, pharmacists have successfully implemented point-of-care diagnostic testing, vaccination programs, and prevention initiatives [5]. These services have been associated with reduced waiting times, higher patient satisfaction, and decreased antibiotic prescribing. Although pharmaceutical care in Poland is at an earlier stage of development, the findings of this study provide practice-based support for regulatory and policy initiatives aimed at formally expanding pharmacists’ competencies.

From the patient’s perspective, pharmaceutical counseling offered several advantages, including accessibility without prior appointment, time savings, rapid symptom relief, and a sense of professional support. Patients in this study emphasized the clarity of communication and pharmacists’ professionalism, which contributed to high adherence and satisfaction [16]. Building patient trust in pharmacists as healthcare providers is crucial, not only for acute conditions but also in preventive care, chronic disease management, and counteracting health misinformation [17,18].

Nevertheless, several limitations should be acknowledged. The observational design without a control group limits the ability to attribute improvements exclusively to the intervention, although the magnitude of reported effects exceeds typical placebo responses. Furthermore, outcomes were assessed subjectively through patient self-report, without objective clinical measures such as reduction in pharyngeal inflammation. Despite these limitations, the study offers valuable evidence supporting the integration of pharmaceutical counseling into sore throat management and, by extension, other self-limiting conditions.

Future research should include randomized controlled trials and cost-effectiveness analyses to further substantiate the value of this model of care [19,20]. Importantly, our study did not directly measure antibiotic use following the pharmacy visit. Subsequent work should quantify post-visit antibiotic initiation, duration, and appropriateness to assess misuse rates and stewardship impact. Additional studies should also explore its application across diverse patient groups, comorbidities, seasonal patterns, and in the management of other minor ailments such as cough, cold, dyspepsia, or mild dermatological conditions. Such evidence would help to consolidate the role of pharmacists in delivering first-contact, evidence-based care that improves patient outcomes while strengthening healthcare system efficiency.

## 5. Conclusions

In this multicenter, prospective, real-world study from Poland, a pharmacist-led sore throat treatment pathway integrating RADT (streptolytic test), structured counseling, and evidence-based symptomatic treatment proved effective, safe, and well-accepted. In the RADT-negative cohort, almost all patients reported improvement and pain relief, without adverse events or dosing errors. This highlights both the clinical benefits and the high quality of the pharmacy service. Although ketoprofen spray was the symptomatic treatment option evaluated, it was only used as an example within the counseling pathway. Therefore, the results support the feasibility and effectiveness of the care model itself and can be transferred to other over-the-counter therapies. At the systemic level, this pathway could help reduce unnecessary consultations and promote rational antibiotic use, strengthening the role of community pharmacies in primary care. Key limitations (observational design, patient-reported outcomes, short follow-up, and symptom scores collected for a single example treatment) limit generalizability. Future work should include comparative or randomized designs, formal cost-effectiveness analyses, and linkage with prescribing/claims data to quantify the impact of post-visit antibiotic use and treatment management across different OTC options and patient subgroups.

## Figures and Tables

**Table 1:** Characteristics of the study group.

	N = 128
Gender	
Male	44 (34.4%)
Female	84 (65.6%)
Age	
Mean (SD)	49.3 (14.9)
Range	19.0–80.0
Median (IQR)	48.5 (24.0)
95%CI	[46.7; 51.9]
Height (cm)	
Mean (SD)	169.8 (9.3)
Range	150.0–198.0
Median (IQR)	168.0 (12.0)
95%CI	[168.2; 171.4]
Weight (kg)	
Mean (SD)	78.1 (16.8)
Range	41.0–165.0
Median (IQR)	78.0 (20.0)
95%CI	[75.2; 81.0]
Place of residence	
Rural area	38 (29.9%)
Small town	44 (34.6%)
Medium city (100–500 thousand inhabitants)	11 (8.7%)
Large city (≥500 thousand inhabitants)	34 (26.8%)
Comorbidities (chronic conditions)	
Yes	52 (40.6%)
No	76 (59.4%)

**Table 2:** Characteristics of the research pharmacist’s assessment of sore throat symptoms (N = 128).

	%
Edema (swelling)	
0—None	1.6
1—Mild	23.4
2—Moderate	50.8
3—Severe	24.2
Redness (erythema)	
0—None	0.8
1—Mild	18.0
2—Moderate	53.9
3—Severe	27.3

**Table 3:** Effectiveness of Ketoprofen Compared to Other Products (N = 128).

Effectiveness Rating	(%)
Better than other products	88.3
Similar	10.9
Worse	0.8

**Table 4:** Onset of Action of Ketoprofen (N = 128).

Speed of Action Rating	(%)
Faster than other medicines (7–10/10)	86.7
Similar	11.7
Slower	1.6

**Table 5:** Degree of Sore Throat Relief Provided by Ketoprofen (N = 128).

Pain Reduction Rating	(%)
Significant pain reduction	75.0
Moderate pain reduction	25.0
No pain reduction	0.0

**Table 6:** Adherence to Dosing Recommendations.

Exceeded Recommended Dose?	(%)
Yes	0.0
No	100.0

**Table 7:** Characteristics of patient satisfaction with the provided service (N = 128).

	%
Satisfaction with advice relative to symptom resolution	
No change	1.6
Improvement	44.8
Marked improvement	53.6
Who should pay for the service	
Patient	7.0
Ministry of Health	50.8
National Health Fund	96.9
Social Insurance Institution	36.7
Supplementary insurance	4.7
Employer	6.3
Pharmaceutical company	26.6
Amount	
PLN 50–60	10.5
PLN 61–70	12.9
PLN 71–80	11.3
PLN 81–90	4.0
Above PLN 100	61.3

## Data Availability

The article presents results in aggregated form. Detailed statistical reports and de-identified raw data are available from the corresponding author.
